# Editorial: Advances in bacteriophage research & development with therapeutic applications

**DOI:** 10.3389/fcimb.2025.1751637

**Published:** 2025-12-10

**Authors:** Mercedes Gonzalez Moreno, Silke Alt, Derry Mercer

**Affiliations:** 1 INCubator for Antibiotic Therapies Europe (INCATE), Basel, Switzerland; 2 Leibniz Institute for Natural Product Research and Infection Biology, Hans Knöll Institute, Jena, Germany; 3 Translational Project Management Office (TPMO), German Center for Infection Research (DZIF), Braunschweig, Germany

**Keywords:** phage therapy, antimicrobial resistance, bacteriophage research, endolysin, artificial intelligence

## Introduction

In response to the global crisis of antimicrobial resistance, phage therapy (PT) is emerging as alternative and complementary therapy to treat bacterial infections. Advances in genomics, synthetic biology and AI play a role in elevating the clinical potential of PT and phage-encoded proteins. The challenge of PT needs to progress beyond compassionate and individual use and address issues including standardized guidelines for phage susceptibility testing, dosing, manufacturing, storage requirements and regulations for clinical trials, to become an established and more broadly used therapeutic option. We are witnessing progress on some aspects, including formation of a working group by the European Committee on Antimicrobial Susceptibility Testing (EUCAST) to develop standardized guidelines for phage susceptibility testing.^1^ Additionally, the European Medicines Agency (EMA) is preparing new guidelines on the development and manufacture of human medicinal products specific for PT.^2^ In 2024 the Portuguese National Authority of Medicines and Health Products, announced the approval of a regulatory framework allowing administration of personalized phage products, following the Belgian model.^3^,^4^ The growing momentum in phage research over the last two decades is contributing to this progress and is paving the way for PT to become a reality.

This Research Topic brings together eleven articles and one correction that collectively contribute to expanding the evidence and understanding of several critical aspects of PT to advance its implementation.

## Structural and functional phage biology

Understanding phage structure, host specificity, lytic mechanisms and the functional role of phage-derived proteins (e.g. endolysins) should contribute to the development of effective phage-based therapies. Three articles in this Research Topic contribute key insights into these crucial aspects.

Khan et al. provides structural analysis of the *Salmonella*-infecting phage Epsilon15, offering detailed characterization of its DNA packaging system and injection apparatus, alongside the functional analysis of its encoded endolysin LysSA05. Bioinformatic and machine learning analyses identified an amphipathic α-helical region within LysSA05 with antimicrobial peptide-like properties, likely responsible for mediating outer membrane interaction and translocation of the catalytic domain to peptidoglycan, a dual function potentially enhancing the enzyme’s efficacy against Gram-negative pathogens. LysSA05 demonstrated antibacterial activity against multidrug-resistant clinical isolates of *E. coli* and *Salmonella* spp., supporting its therapeutic potential.

Ning et al. addressed the evolutionary dynamics of *E. coli* phage BW-1, investigating whether this phage can expand their host range through experimental evolution. Their findings suggest that host-switching is limited, and when adaptation occurs, it is genetically constrained, and may come with fitness costs (infectivity or virulence). The authors suggest that there is a reduced risk of therapeutic phages evolving to infect non-target or beneficial gut bacteria.

Alsaadi et al. performed a prophage analysis of 44 P*. aeruginosa* isolates, revealing high diversity with most prophages classified as novel genera and lacking antimicrobial resistance or virulence genes. Many encoded different anti-phage defence mechanisms. This demonstrates prophage genomics can help to predict resistance trends and optimize treatment strategies.

## Selection, optimization, and production

The success of PT depends on identification of effective phages rational cocktail design, and scalable production and optimization strategies, amongst others. Four articles in this Research Topic highlight advances and challenges in these areas.

Rieper et al. tackled the challenge of phage selection, aiming to determine the best strategies for evaluating their therapeutic potential. Their study compared double agar overlay plaque assay and planktonic killing assay to assess the host range and lytic efficacy of a diverse range of lytic phages against clinical *P. aeruginosa*. Their findings highlight the need for standardised and rigorous testing protocols and the integration of *in silico* tools for optimal phage selection.

Doud et al. offer a forward-looking perspective, exploring how AI can be leveraged to improve PT. The authors discuss recent AI advances that enable the prediction of phage-host compatibility, discovery of functional phage genes, and rational engineering of synthetic phages, but emphasize the need for experimental validation.

Ramirez-Garcia et al. present a novel antimicrobial approach base on P4 and P2 bacteriophages engineered as phage-like transducing particles that carry a genetically encoded multi-lysin cassette instead of viral DNA. These virus-free particles cannot replicate and offer controllable expression of lytic proteins inside bacteria. The authors achieved high-yield production through bioprocess optimization and showed antimicrobial activity against *E. coli* in pure culture and a co-culture infection model.

Kulkarni et al. contribute a perspective that proposes synthetic cells as programmable and modular platforms for bacteriophage production, control and delivery, moving beyond the constraints of traditional bacterial hosts. The authors further envision the integration of these synthetic cells within smart antimicrobial materials, and outline the direction toward AI and microfluidics-driven platforms that could enable predictive phage–host modelling, high-throughput screening and personalized PT workflows.

## Preclinical and clinical applications

Current knowledge gaps exist on different clinical aspects in PT such as appropriate dosage, route and frequency of administration, concomitant use of antibiotics, phage resistance, immunogenicity and phage pharmacokinetic properties to better guide clinical implementation. Together, three studies in this Research Topic offer a view of PT’s translational journey.

Zhu et al. assessed the combined efficacy of the lytic phage HZJ31 with tigecycline against carbapenem-resistant *K. pneumoniae in vitro* and in *Galleria mellonella* larvae, demonstrating synergistic effects, significantly enhancing bacterial suppression and improving survival outcomes *in vivo*. Characterization of phage-resistant bacteria revealed trade-offs leading to reduced virulence and increased sensitivity to piperacillin and gentamicin, supporting the potential of phage-antibiotic combination therapies.

Peez et al. investigated the application of phage in a sheep model of fracture-related infection caused by *S. aureus*, comparing intravenous versus local phage delivery. This study revealed key pharmacokinetic aspects in this preclinical model, especially the challenges of phage neutralization and rapid bloodstream clearance, that can limit therapeutic efficacy.

Wahl et al. reported a clinical case of co-administration of phages for successful antibiotic suppression therapy in a periprosthetic hip and knee joint infection caused by MRSA. Infection persistence led to consideration of PT. Successful outcomes illustrated PT as a valuable adjunct to suppress infection but exposed the logistical challenges in facilitating therapy.

## Regulatory perspectives

The increasing body of clinical data on PT is driving progress in adapting regulatory frameworks and creating new pathways that address specific characteristics of phage therapeutics.

Fürst-Wilmes et al. provided a comprehensive overview of the current regulatory landscape for PT in Europe, highlighting key developments on manufacturing standards, quality criteria and adaptations of pharmaceutical legislation to PT.

## Conclusions

Collectively, these articles reflect the diverse and rapidly advancing landscape of PT and underscore the multidisciplinary and collaborative effort needed to bring it to clinical reality.

## Footnotes

1 https://www.eucast.org/ast-of-phages

2 https://www.ema.europa.eu/en/development-and-manufacture-human-medicinal-products-specifically-designed-phage-therapy-scientific-guideline

3 https://www.infarmed.pt/documents/15786/9697484/2024-11-15_Delibera%C3%A7%C3%A3o_112_CD_2024/d0424242-a103-1256-cd7d-526aaf12dfac

4 Jean-Paul Pirnay, Gilbert Verbeken, Magistral Phage Preparations: Is This the Model for Everyone?, Clinical Infectious Diseases, Volume 77, Issue Supplement_5, 1 November 2023, Pages S360–S369, https://doi.org/10.1093/cid/ciad481

